# Associations between epigenetic age and brain age in young people

**DOI:** 10.1038/s41598-025-11350-x

**Published:** 2025-07-22

**Authors:** Faye Sanders, Vilte Baltramonaityte, Gary Donohoe, Neil M Davies, Erin C. Dunn, Charlotte A. M. Cecil, Esther Walton

**Affiliations:** 1 Department of Psychology, University of Bath, Claverton Down, Bath, BA2 7AY UK; 2 School of Psychology, University of Galway, Galway, Ireland; 3 Centre for Neuroimaging, Cognition & Genomics, University of Galway, Galway, Ireland; 4 Division of Psychiatry, University College London, London, UK; 5 Department of Statistical Science, University College London, London, UK; 6 Department of Public Health and Nursing, Norwegian University of Science and Technology, Trondheim, Norway; 7 Department of Sociology, College of Liberal Arts, Purdue University, West Lafayette, IN USA; 8 Psychiatric and Neurodevelopmental Genetics Unit, Centre for Genomic Medicine, Massachusetts General Hospital, Boston, MA 02114 USA; 9 Department of Psychiatry, Harvard Medical School, Boston, MA 02115 USA; 10 Department of Child and Adolescent Psychiatry/Psychology, Erasmus MC, University Medical Center Rotterdam, Rotterdam, the Netherlands; 11 Department of Epidemiology, Erasmus MC, University Medical Center Rotterdam, Rotterdam, the Netherlands; 12 Molecular Epidemiology, Department of Biomedical Data Sciences, Leiden University Medical Center, Leiden, the Netherlands

**Keywords:** Epigenetic age, Brain age, Adolescence, ALSPAC, Development, DNA methylation, Epigenetics, Cognitive ageing

## Abstract

Recent research suggests biological age, based on epigenetic or neuroimaging measures, may predict health traits in adulthood more accurately than chronological age. However, it is unclear if these findings apply earlier in life. We aimed to characterise the performance and interdependence between measures of biological age in young people, leveraging a longitudinal subsample from the population-based ALSPAC cohort (*n* = 386). We derived four epigenetic age measures from blood samples in young people (17–19 years) and a measure of brain age derived from structural neuroimaging data (18–24 years). We examined associations between measures of biological age, and relationships with five measures of physical, cognitive and mental health (8–18 years). We found little evidence for an association between brain age and epigenetic age measures, after accounting for age, sex, cell type, array and study (beta range: -0.59 to 0.59, all *p* > 0.05). Increased smoking_DNAm_ was associated with advanced epigenetic age (PACE and Zhang clock), and increased BMI_*sds*_ with advanced EpiAge_Horvath(diff)_ (all *p* < 0.05), but not brain age. Depressive symptoms and cognitive ability were unrelated to all measures of biological age. Our findings highlight the variability of epigenetic- and brain-based age measures in young people, emphasizing the importance of tracking ageing in younger populations.

**Supplementary Information:**

The online version contains supplementary material available at 10.1038/s41598-025-11350-x.

## Introduction

Longer life expectancy and the associated increases in age-related illnesses are placing growing pressures on society. Although the risk of developing physical or mental illness increases generally with age, there is a large amount of variability within individuals of the same chronological age. Recent research suggests that defining age through *biological measures*, rather than self-reported *chronological measures*, may provide a more accurate predictor of health outcomes. Measures of biological age can be derived from various biological data, including epigenetic (i.e. DNA methylation) signatures and structural brain scans^1,2^. Advanced biological age has been associated with several health outcomes, such as cognitive decline, health span, and all-cause mortality^3–5^.Therefore, analytic approaches that incorporate measures of biological age, as substitutes or in addition to chronological age, may improve our ability to predict mortality and age-related diseases^6^.

A growing body of research focuses on estimates of biological age based on DNA methylation profiles, known as epigenetic age (or epigenetic ‘clocks’). Numerous epigenetic age measures have been developed (here referred to as e.g. *EpiAge*_*Horvath*_, EpiAge_Zhang_). Epigenetic age profiles consistently correlate with chronological age across various tissues, cell types and species^2^. Epigenetic age has been found to be an important predictor of mainly physical, but also some mental and cognitive health traits, especially in older individuals^7,8^. For example, advanced epigenetic age was closely associated with increased BMI, frailty and all-cause mortality in adults^6,9^. Critically, chronological age no longer significantly predicted health outcomes in both adults and elderly people^6^ when epigenetic age was taken into account. Thus, these findings indicate the predictive potential of epigenetic age for health outcomes over and above chronological age.

However, most of these epigenetic age measures are derived from easily accessible peripheral tissues such as blood and little is known about how these peripheral age-related measures relate to ageing processes in the brain. There is some weak, albeit consistent, evidence that biological ageing is a system-wide process, associated with a decline across tissues^10–12^. For example, Hillary et al.^11^ found advanced epigenetic age (EpiAge_GrimAge_) associated with decreased brain volume and increased brain white matter hyperintensities in an elderly sample of 540 participants. Similarly, Whitman and colleagues^12^ found that epigenetic rate of ageing (EpiAge_PACE_) associated with decreased total brain volume in middle-aged adults. Gadd and colleagues^13^ reported that epigenetic scores for smoking correlated across blood and brain tissue, also indicating that some epigenetic measures follow a system-wide pattern across the blood and brain. Hoare and colleagues^14^ found advanced epigenetic age (EpiAge_Horvath_ and EpiAge_Hannum_) associated with alterations in cortical thickness and surface area across some brain regions in adolescents from low-income households.

Conversely, there is also evidence for substantial variability in the rate of functional and structural decline across different tissues, a concept referred to as the ‘mosaic of ageing’^15,16^. If temporal and cross-tissue variability exists in ageing, there may be little consistency between peripheral and brain-based ageing processes. Clarifying the nature and direction of associations between cross-tissue measures of age is important, as it could increase our understanding of the mechanisms and interactions within the ageing process across the lifespan and within specific developmental stages.

The recent development of two new brain-based measures of age might enable researchers to address this question. First, Shireby and colleagues^17^ developed a ‘cortical clock’ (EpiAge_Cortical_ ) - a novel epigenetic age measure based on post-mortem cortical tissue across the life course, which also performs well when applied to blood tissue. Second, a neuroimaging measure of brain age has been created^1^. This neuroimaging measure is based on structural magnetic resonance imaging (MRI) data, including grey matter volume, cortical thickness, and surface area. Akin to the role of epigenetic clocks in predicting health outcomes, advanced brain age has been associated with age-related illnesses such as Alzheimer’s disease and mild cognitive impairment^18,19^. Strikingly, an association between brain age and illnesses previously less defined as related to chronological age (e.g., depression, schizophrenia and epilepsy) has also been observed^1,20,21^, suggesting that the biological process of ageing may play a greater role in the development or symptomology of these illnesses than previously considered.

To our knowledge, only a few studies have tested for associations between epigenetic age and brain age. In a cohort of 620 older adults, Cole et al.^22^ found little evidence that epigenetic age (EpiAge_Horvath_) correlated with brain age. However, the authors did report independent associations between mortality risk and epigenetic age or brain age, respectively, highlighting the unique importance of both measures for healthy ageing. Similar results were reported by Zheng et al.^23^ in a sample of 326 adults. Teeuw et al.^24^ also found little evidence that two measures of epigenetic age (including EpiAge_Horvath_) associated with brain age in 172 adults with and without schizophrenia. An additional study, using genetic data, reported low genetic correlations between different measures of epigenetic age and brain age^25^. Mareckova et al.^26^ found weak correlations between epigenetic age (EpiAge_Horvath_) and brain age in young adults. These correlations varied in strength and direction, and across age groups, suggesting there may be more complex and developmentally patterned relationships.

So far, it is unclear to which degree epigenetic age and brain age associate with each other in younger samples. Studies of children and adolescents have detected associations between *either* epigenetic *or* brain age and markers of development, behaviour or health^27,28^. However, research to date has not yet investigated the degree to which epigenetic and brain age associate *with each other* in population-based samples of young people. Knowing the relationship between these markers is critical to understand the interdependence of different biological ageing processes and how to design early and effective interventions to prevent advanced ageing.

The aim of this study was threefold:

Aim 1) to estimate the associations between chronological and biological age (including EpiAge_Horvath_^2^, EpiAge_Zhang_^29^, EpiAge_Cortical_^17^,EpiAge_PACE_^30^ and BrainAge^31^) in a population-based cohort of young people;

Aim 2) to examine the associations across modalities of biological age, (i.e., measures of epigenetic age and brain age, which were developed in either peripheral or cortical tissues); and.

Aim 3) to determine the associations between these biological age measures and physical, cognitive and mental health traits.

## Results

Our final analytic sample consisted of *n* = 386 young people (Table 1).

**Table 1:** Sample demographics. DNAm = DNA methylation, mri = magnetic resonance imaging, CRP = C reactive protein, bmi = body mass index (standard deviation score), Smoking_DNAm_ = cg05575921 epigenetically predicted smoking behaviour, and WISC = Wechsler Intelligence Scale for Children.

	Overall (*N* = 386)
Sex	
Females	53 (13.7%)
Males	333 (86.3%)
Ethnicity	
White	386 (100%)
Age at DNAm (years)	
Mean (SD)	17.6 (0.575)
Age at MRI (years)	
Mean (SD)	19.8 (1.16)
CRP at 17y (mg/L)	
Mean (SD)	0.956 (1.14)
Missing	18 (4.7%)
BMI at 17y (SD score)	
Mean (SD)	0.32 (1.09)
Missing	11 (2.8%)
Smoking_DNAm_ at 17y	
Mean (SD)	0.838 (0.0506)
Cognitive performance at 8y (WISC)	
Mean (SD)	105 (16.7)
Missing	29 (7.5%)
Depression score at 17y	
Mean (SD)	0.281 (0.763)
Missing	27 (7.0%)
DNAm array	
450k	153 (39.6%)
EPIC	233 (60.4%)
MRI study sample	
study1	55 (14.2%)
study2	32 (8.3%)
study3	299 (77.5%)

### Biological ages capture chronological age to varying degrees in young people

**Fig. 1:**
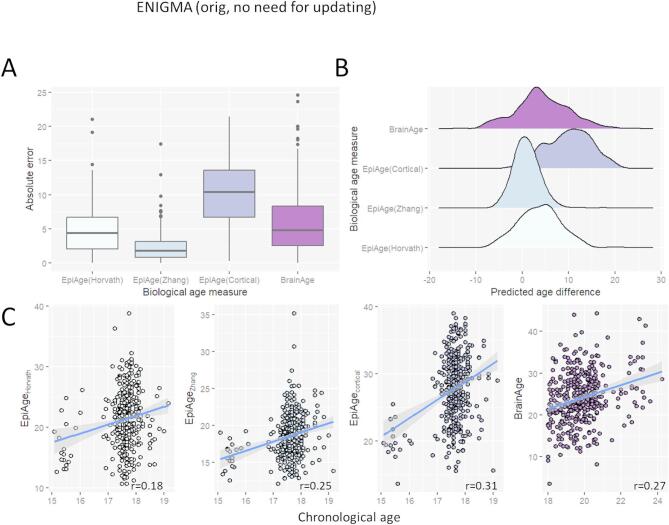
(**A**) Mean absolute errors, (**B**) predicted age differences and (**C**) age correlations across four state measure of biological age.

The accuracy of four biological age predictors was acceptable with a mean absolute error (MAE) between 2.2 and 5.9 years (range: 0.0 to 24.5 years; Fig. 1A), which is comparable to other studies using this data or similar^32–35^. The accuracy of EpiAge_Cortical_ was poor (MAE calculated in relation to chronological age = 10.2 years; see also Table S1), but also comparable to other studies^36,37^. Predictions were all slightly biased towards overestimating ages (Fig. 1B and Table S1), likely due to regression towards the mean, as is seen elsewhere in testing samples with younger ages than the training sample^38^. The smallest average overestimation was observed for EpiAge_Zhang_ (diff = 0.9y), followed by EpiAge_Horvath_ (diff = 3.7y), BrainAge (diff = 4.3y) and EpiAge_Cortical_ (diff = 10.1y). That is, even though the MAE for most predictors were acceptable, age predictions were generally ‘older’ than chronological age.

**Fig. 2:**
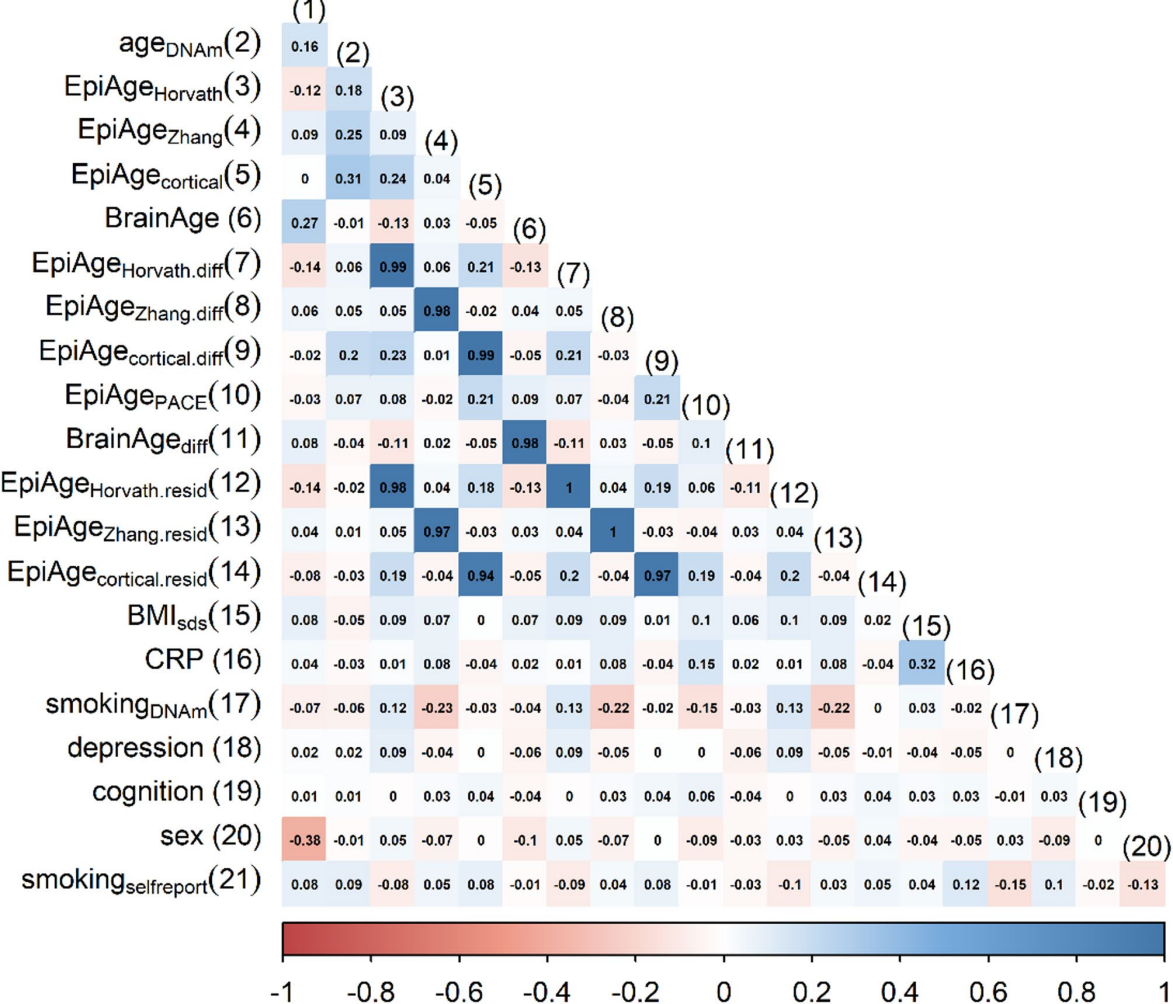
Heatmap showing Pearson correlations across all measures. Measure^1^ – shown on the top diagonal by number only, refers to age_MRI_.

Predicted ages correlated positively, but not strongly with chronological age, which aligns with a previous study using this data^39^ and is unsurprising given the narrow age range of this sample (Figs. 1C and 2). The strongest Pearson correlations were observed for EpiAge_Cortical_ (*r* = 0.31), followed by BrainAge (*r* = 0.27), EpiAge_Zhang_ (*r* = 0.25), and EpiAge_Horvath_ (*r* = 0.18). These results were robust to outliers, as similar patterns were observed using Spearman correlations (Figure S1).

As expected, biological age *residuals* were unrelated to chronological age (r range: -0.03 to 0.01, Fig. 2). However, biological age *difference* scores were also largely unrelated to chronological age (r range: 0.05 to 0.08), except for EpiAge_Cortical(diff)_ (*r* = 0.20). Because difference scores correlated strongly with residual scores (all r’s > 0.97), we focused on difference scores (and EpiAge_PACE_), while presenting results for residual scores throughout the supplemental materials.

### Little evidence for an association between brain age and epigenetic ages in young people

Although some biological age measures or their difference scores were correlated with each other (range r: -0.13 to 0.24; Fig. 2, Figures S1 and S2), most associations decreased substantially once controlling for batch, sex, cell type, array or age (and MRI study when BrainAge_(diff)_ was the outcome; Table S2 and S3). The exception was Horvath’s epigenetic age and age difference score, which were associated positively with all measures of cortical epigenetic age (Table S2). These findings do not provide convincing evidence that measures of epigenetic or brain age are strongly related to each other in young people.

Interestingly, despite being trained on cortical tissue, all measures of cortical epigenetic age were more strongly associated with other epigenetic age measures in blood tissues (r between − 0.13 and 0.24) than with measures of brain age (r between − 0.05 and 0.06). This finding could suggest that biological modality of data collection (epigenetics versus neuroimaging) might account for more heterogeneity than the tissue of interest (blood versus brain).

**Fig. 3:**
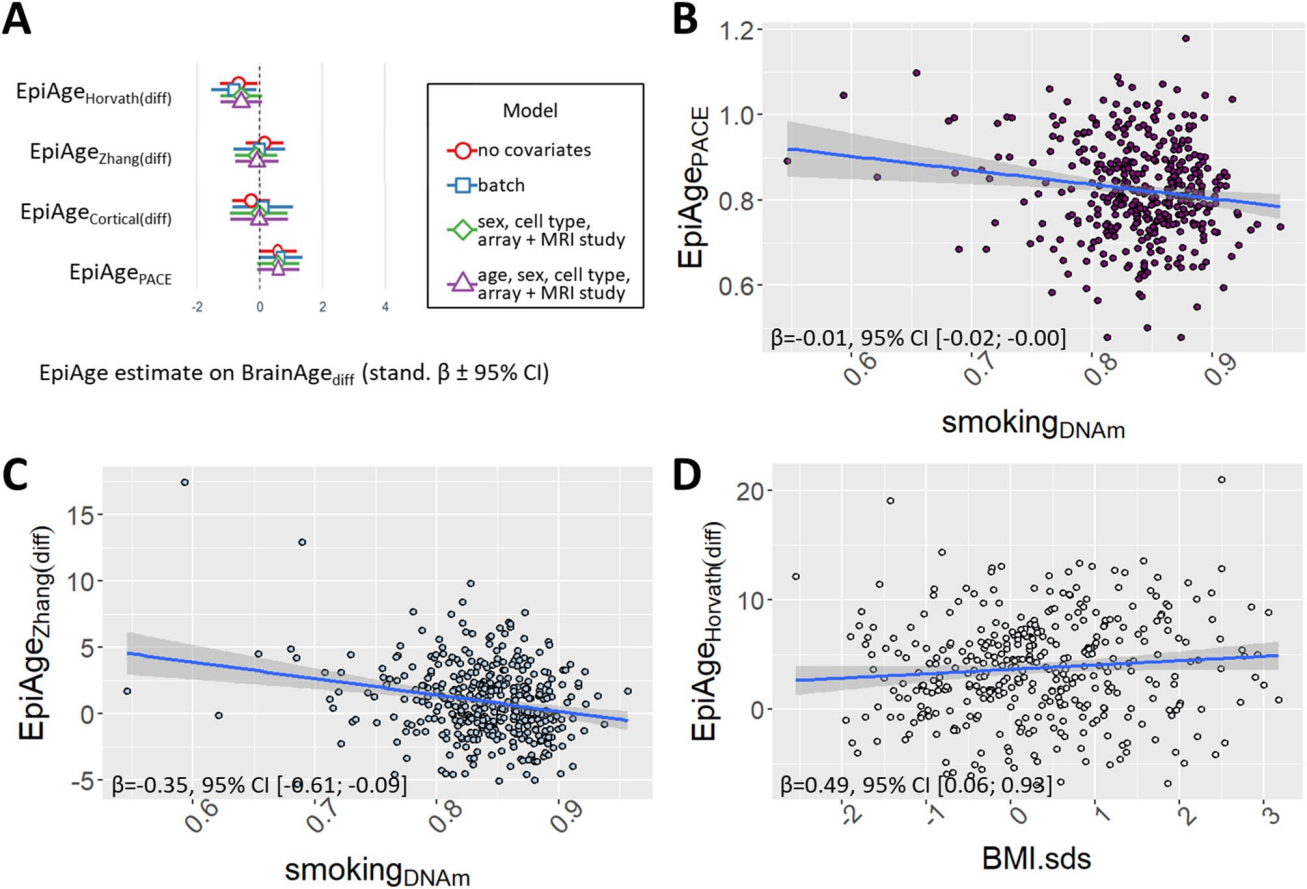
(**A**) Standardized regression betas (95% CI) of four epigenetic age difference scores predicting BrainAge_(diff)_. (**B**-**C**) Smoking_DNAm_ behaviour and (**D**) BMI_*sds*_ associated with three measures of epigenetic age. Due to the low number of participants who self-reported on their smoking (22%), we used a measure of epigenetically predicted smoking_DNAm_ (based on DNAm_cg05575921_), a well-validated proxy for smoking. Lower smoking_DNAm_ values associate with increased smoking behaviour.

BrainAge_(diff)_ remained largely unrelated to the other epigenetic_(diff)_ scores (Fig. 3A and Table S3). Although overall largely insignificant, most associations between BrainAge_(diff)_ and state-based epigenetic age measures were negative, whereas the association with EpiAge_PACE_, a rate-based measure, was positive. No influential outliers or a violation of homoskedasticity was detected. Principal component analyses indicated no clustering of participants based on their biological ages or difference scores (Figure S3A-C), but 82% of participants showed consistent accelerated (or decelerated) biological age in more than one measure (Figure S3D).

### Epigenetically-predicted smoking behaviour and BMI_sds_ associated with blood- but not brain-related measures of age

A faster rate of ageing (EpiAge_PACE_) and advanced epigenetic age (EpiAge_Zhang(resid/diff)_) were linked to increased smoking_DNAm_ (beta_std_ between − 0.01 and − 0.35; Fig. 3B-C and Table S4; note that lower smoking_DNAm_ values associate with increased smoking behaviour), even after controlling for age, sex, array, MRI study and cell type. EpiAge_Horvath(resid/diff)_ were positively associated with BMI_*sds*_ (beta_std_ = 0.49; Fig. 3D and Table S4). No influential outliers or violations of homoskedasticity were detected.

All remaining associations between biological ages (including all difference or residual scores of epigenetic or brain age) and measures of physical (CRP), cognitive or mental health (cognition, depression) were weak and insignificant (standardized regression betas ranged between − 0.36 and 0.28; Table S4).

A similar pattern of results was obtained when using the Centile measure of BrainAge_(diff)_ (Figures S4, S5 and Table S5).

## Discussion

In this study, we characterised the association between epigenetic age and brain age in young people. We highlight three key findings: (1) multiple measures of biological age captured chronological age in young people; (2) there was little evidence that measures of epigenetic age and brain age were related and (3) smoking_DNAm_ and BMI_*sds*_ predicted advanced epigenetic age, but not brain age.

First, chronological age was associated with biological age (including both epigenetic and brain age) to varying degrees in young people. With an age correlation ranging from 0.18 to 0.31 and an MAE ranging from 2.2 to 10.2 years, overall biological age measures were moderate-to-good indicators of chronological age in young people. We note, however, that biological age predictors, on average, overestimated chronological age by 1 to 10 years. Although large, the range of errors is consistent with previous research^35,40,41^. Sample demographic and methodological differences may explain some of these large MAEs. For example, the largest MAE was observed for EpiAge_Cortical_, which was developed in cortical tissues on a smaller training sample and had lower accuracy when applied to non-cortical tissues such as blood^17^. Conversely, the lowest MAE was observed for EpiAge_Zhang_, which was developed in a large training sample and based on blood and saliva tissue. These results further suggest that tissue and training sample size are important factors affecting prediction accuracy. Furthermore, the median age in the training sample for EpiAge_Cortical_ was 57 years – over 15 years older than the median age of the training samples used for EpiAge_Horvath_.

Despite these caveats, we observed consistency across measures. Most people who showed advanced age in one measure also did so in other measures. Furthermore, as argued elsewhere^42^, performance metrics such as MAE are sample-specific (i.e., they cannot be easily compared across studies) and should be considered in combination with other metrics such as correlation coefficients. For example and similar to our study, large increases in MAE have been reported despite acceptable correlations (*r* > 0.5) between chronological and predicted age, especially in situations where the age range of the testing sample was very narrow and different to that of the training sample^42^. In line with this, we observed that – even though EpiAge_Cortical_ had the largest MAE across all biological ages – it was also the measure most strongly associated with chronological age and Horvath’s epigenetic age. Hence, a large MAE should not be considered the only indicator for model accuracy.

While these performance metrics help assess model fit, they do not explain what drives individual variability in biological ageing. The large predicted age differences reported in our study may indicate the extent to which biological age can diverge from chronological age in young people, possibly due to genetic factors, environmental variation in childhood experiences, or stochastic effects. Previous research has reported decelerated epigenetic age clustering within families^43^ and a partial genetic basis underlying some, but not all measures of epigenetic age^44^ and brain age^45^. Other research has highlighted the impact of environmental factors such as socioeconomic status and childhood adversity in predicting advanced epigenetic age^35,46^. Further research is needed to understand the ageing process – and factors that influence this process - during the first two decades of life to create interventions that can reduce ageing-related health inequalities seen in later life^47^.

The second key finding is that there was little evidence for an association between brain age and epigenetic ages in young people. This interpretation is consistent with Teeuw et al.^24^, Zheng et al.^23^ and Cole et al.^22^, whereby only weak associations were detected between brain age and epigenetic age in a sample of younger or older adults. Our findings, however, contradict previous research in older individuals, which reported associations between neuron density or brain vascular lesions, as measures of brain age, and whole-brain functional connectivity and epigenetic age^11,26,48,49^.

A moderate sample size or small differences in age at assessment at blood draw and MRI could be one explanation for the lack of associations. However, research into the blood-brain barrier may provide an alternative insight into some of these inconsistencies. The blood-brain barrier is most intact in early development and only degrades in later life, resulting in increased permeability of the blood-brain barrier with age^50^. This increased permeability could affect neuron density^51^, the cellular measure of brain age used in Lu et al.^48^, but might relate less strongly to the brain imaging measure of brain age used in the current study. Hence, it is possible that epigenetic age associates with microscopic (cellular), rather than macroscopic measures of brain age, especially in later life, when the blood-brain barrier may be less intact^50^.

The third key finding indicated that advanced biological age in young people is only weakly linked to health traits. Of all tested traits, smoking_DNAm_ behaviour was most consistently associated with measures of epigenetic age, including the pace of epigenetic ageing, but not with brain age. The association with epigenetic age is consistent with previous research, as smoking has been associated with advanced epigenetic age, specifically in lung tissue^52^. The lack of association between smoking_DNAm_ and brain age however appears to be inconsistent with previous findings, whereby brain volume loss or brain age has been reliably associated with smoking in middle-aged adults^53–55^. It is possible that effect of environmental exposures such as smoking on the brain do not become apparent until later in life. Additionally, duration of exposure could be relevant, as young people will have smoked on average for a shorter period than adults, limiting the time for smoking to reliably affect tissues more distal from the lungs^56^. Finally, methodological considerations may also contribute to the lack of association between smoking and brain age. Although we used a validated biomarker of smoking, this measure is derived from epigenetic (DNA methylation) data. This may lead to a stronger association of smoking_DNAm_ with epigenetic age as opposed to brain age.

Our study should be considered in light of the following limitations: the age range of our sample was very narrow, making it challenging to distinguish between measurement error and true predicted age deviations. However, all measures of biological age were associated with chronological age despite the narrow age range and similar to estimates reported previously^32–36^. Second, our sample was limited to the period of late adolescence to young adulthood. Biological age measures may associate differently with each other or health traits at different developmental periods. However, using a sample with a highly constrained age range allowed us to derive developmentally specific conclusions. It might also have minimised some of the noise that could have obscured an association in studies with a larger age range. Third, the sample size – although similar to that of previous studies – was moderate, limiting power, and restricted to individuals of white ethnicity. Future research is needed to replicate our results in larger, more ethnically diverse samples of individuals across a wider age range, to ensure that our findings can be more generalisable outside of this study population.

In summary, we investigated the association between chronological age and two modalities of biological age – specifically measures of epigenetic and brain age, in young people. The variability between these age measures in young people highlights the dynamicity of ageing across the lifespan and signifies the importance of tracking the mosaic of ageing in younger populations to inform prevention targets to support physical and mental health across the life course.

## Methods

### Study population

Our sample was drawn from the Avon Longitudinal Study of Parents and Children (ALSPAC). Pregnant women resident in Avon, UK with expected dates of delivery between 1st April 1991 to 31st December 1992 were invited to take part in the study. The initial number of pregnancies enrolled was 14,541 (for these at least one questionnaire has been returned or a “Children in Focus” clinic had been attended by July 19 1999). Of these initial pregnancies, there was a total of 14,676 foetuses, resulting in 14,062 live births and 13,988 children who were alive at 1 year of age^57–59^. Please note the study website contains details of all the data that is available through a fully searchable data dictionary and variable search tool (http://www.bristol.ac.uk/alspac/researchers/our-data/). Ethical approval for the study was obtained from the ALSPAC Ethics and Law Committee (20–195) and the Local Research Ethics Committees. Consent for biological samples has been collected in accordance with the Human Tissue Act (2004).

For this study, we restricted our analyses to young people with both methylation and brain imaging data, as described in the next sections. Due to very small numbers of parents who self-reported to be of Caribbean, African, Indian, Chinese or ‘other’ ethnicity (< 5), only young people whose parents self-identified as White were included in the analyses sample. To avoid potential biases arising from shared family environment, we only included the first-born twin per family.

### Methylation preprocessing

Blood samples from young people at age 17 years were selected for analysis as part of the Accessible Resource for Integrative Epigenomic Studies (ARIES, http://www.ariesepigenomics.org.uk/;^60^). Bisulfite conversion was performed with the EZ-96 DNA methylation kit (shallow; Zymo Research Corporation, Irvine, USA). DNA methylation levels were then measured on two arrays: the Illumina Infinium HumanMethylation450 and EPIC BeadChip array (Illumina Inc., San Diego, USA). Preprocessing in ALSPAC was performed with the meffil package, using functional normalization.⁠ Quality control checks included mismatched genotypes, mismatched sex, incorrect relatedness, low concordance with other time points, extreme dye bias, and poor probe detection.

We derived a comprehensive list of epigenetic age measures in young people. Specifically, we obtained state measures of epigenetic age based on:

- Horvath et al.^2^: EpiAge_Horvath_
- Zhang et al.^29^: EpiAge_Zhang_
- Shireby et al.^17^: EpiAge_Cortical_ and
- Belsky et al.^30^: EpiAge_PACE_

For each state measure, we derived a predicted age *difference* (diff) score, which equalled epigenetic age minus chronological age. We also derived a predicted age *residual* (resid) score, commonly reported and obtained by regressing chronological age on epigenetic age. Residual scores can be interpreted as the biological age of a person, which is independent of chronological age. Despite residual scores being more commonly used in epigenetic research, we present difference scores in our main analyses, as these are more closely harmonized with our measure of brain age (where it is convention to use difference scores). Results using residual scores are presented in the supplementary materials. Models using difference scores adjusted for chronological age (i.e. model 4, see ‘Statistical analysis’ section) will approximate models using residual scores.

Because our analyses were based on participants younger than 18 years, we did not derive epigenetic age measures which were trained exclusively on adult samples^6,40^. We also created a Pace of Ageing Epigenetic (DunedinPACE;^30^) score, which is a rate- rather than a state measure of epigenetic age. Although trained on adults, we included DunedinPACE to be inclusive of both state- and rate-based measures. Whilst conceptually and methodologically different from the other epigenetic age measures, for clarity we refer to this measure as EpiAge_PACE_ here to clarify that this is also an epigenetics-based measure. Residual and difference scores were not produced for EpiAge_PACE_ as it is a rate-based measure. In total, we created seven age measures using the dnaMethyAge package (https://github.com/yiluyucheng/dnaMethyAge) using default settings. Data was quantile normalized when calculating EpiAge_Horvath_. Of the 1183 unique CpG sites across these age measures, we had 20 missing probes, which were imputed with the mean of the reference dataset by default. For an overview of measures, see Table S6.

### MRI preprocessing

Between ages 18 to 24 years, a subset of ALSPAC participants were invited to three separate ALSPAC neuroimaging studies (Sharp et al., 2020). In total, MRI data was acquired for *n* = 958 participants, of which *n* = 386 also had methylation data after quality control. For each participant, structural neuroimaging data were acquired on a General Electric 3T HDx scanner. T1-weighted images were processed using the automated FreeSurfer brain imaging software package (Version 6.0.0). Reconstructed images were subjected to quality control measures following the ENIGMA consortium structural image processing protocol (http://enigma.ini.usc.edu/protocols/imaging-protocols/ and Sharp et al., 2020). From these data a measure of brain age was derived in early adulthood (mean = 19.8y; SD = 1.2y) using ridge regression combining seven subcortical regions, lateral ventricles, and thickness and surface area for 34 bilateral cortical regions^1^. We derived a difference score (Brain_(diff)_) but not a residual score, as the accepted standard in the field is to describe advanced brain age through a difference score^61^. In a sensitivity analysis, we validated our results using a different brain age measure (CentileBrain;^62^).

### Health phenotypes

Biological age has been linked to both physical, cognitive and mental health outcomes in adulthood and old age^1,8,63^. To assess these relationships in young people, we selected five key measures:^1^ C-reactive protein levels as a measure of inflammation (CRP, in mg/L measured at age 17y);^2^ sex- and age-adjusted BMI (BMI_sds_, measured at age 17y; adjusted using the *childsds* R package and *uk.who* reference);^3^ smoking_DNAm_ behaviour;^4^ cognitive performance (WISC performance test score, only available at 8 years); and^5^ self-reported depressive symptoms (based on CIS-R measured at 17.5y). Due to the low number of participants who self-reported on their smoking (43% missing), we used a measure of epigenetically predicted smoking_DNAm_ (based on DNAm_cg05575921_), a well-validated proxy for smoking^64^. In those who did self-report, epigenetically predicted smoking successfully differentiated between smokers and non-smokers (Figure S6).

### Statistical analysis

**Fig. 4:**
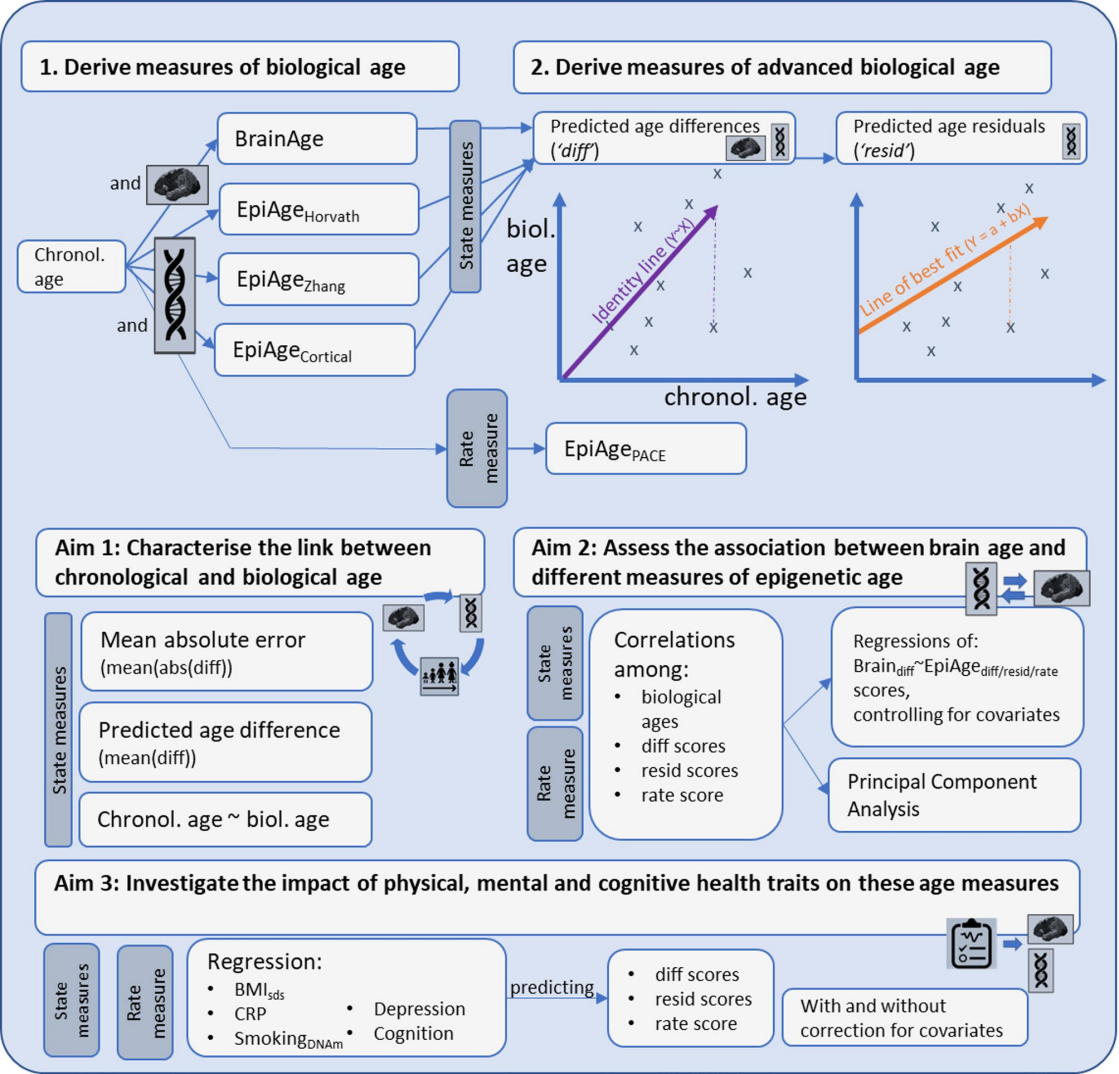
Analysis flow chart.

Figure 4 displays our analysis flowchart. For Aim 1 and 2, we first assessed the mean deviation and MAE between chronological and biological age. Larger deviation or MAE values indicate a larger disparity between chronological and biological age. We then used Pearson correlations to assess the association between chronological age, biological ages, their difference and residual scores. To assess the robustness of these correlations against extreme values, we also applied Spearman rank correlations. We then used principal component analysis to characterise any potential clustering of participants based on their biological age measures, and linear regression to assess the relationships between biological age measures in four regression models with different covariate adjustments:

- Model 1: no adjustment;
- Model 2: controlling for possible batch effects;
- Model 3: controlling for sex, array (450k versus EPIC), MRI study, and cell type (based on Reinius et al. (2012); and.
- Model 4: model 3 + age.

Model 4 was carried forward to investigate the relationship between biological ages and health phenotypes (Aim 3). As health traits were obtained either at the same time or earlier than the biological data, health traits were modelled as predictors of biological age. To minimise the influence of extreme outliers, (1) all measures of biological age and health were winsorized based on a cut-off of 5 times the interquartile range (effectively, this replaced one value for EpiAge_Zhang(resid)_ and 17 values for CRP (4.6%), and (2) regression outputs were inspected for influential outliers using Cook’s distance. Inferences were made based on the size and confidence interval of effects.

## Electronic supplementary material

Below is the link to the electronic supplementary material.

Supplementary Material 1

## Data Availability

ALSPAC data access is through a system of managed open access. The steps below highlight how to apply for access to the data included in the data note and all other ALSPAC data:1.Please read the ALSPAC access policy ( http://www.bristol.ac.uk/media-library/sites/alspac/documents/researchers/data-access/ALSPAC_Access_Policy.pdf) which describes the process of accessing the data and samples in detail, and outlines the costs associated with doing so.2.You may also find it useful to browse our fully searchable research proposals database (https://proposals.epi.bristol.ac.uk/?q=proposalSummaries), which lists all research projects that have been approved since April 2011.3.Please submit your research proposal ( https://proposals.epi.bristol.ac.uk/) for consideration by the ALSPAC Executive Committee. You will receive a response within 10 working days to advise you whether your proposal has been approved.
